# The detection of oral pre- malignant lesions with an autofluorescence based imaging system (VELscope^TM^) – a single blinded clinical evaluation

**DOI:** 10.1186/1746-160X-9-23

**Published:** 2013-08-23

**Authors:** Henning Hanken, Juliane Kraatz, Ralf Smeets, Max Heiland, Marco Blessmann, Wolfgang Eichhorn, Till Sebastian Clauditz, Alexander Gröbe, Andreas Kolk, Madiha Rana

**Affiliations:** 1 Department of Oral and Maxillofacial Surgery, University Medical Center Hamburg-Eppendorf, Martinistraße 52, 20246 Hamburg, Germany; 2 Institute of Pathology, University Medical Center Hamburg-Eppendorf, Hamburg, Germany; 3 Department of Oral and Maxillofacial Surgery, Klinikum rechts der Isar, Technische Universität München, Munich, Germany; 4 Department of Differential Psychology and Psychological Assessment, Helmut-Schmidt-University, University of the Federal Armed Forces, Hamburg, Germany

**Keywords:** Oral cancer, Autofluorescence, VELscope^®^, Early cancer detection

## Abstract

**Objective:**

The disease specific five-year survival rate especially for patients with advanced oral cancer has not improved significantly over the period of time. The most effective way of combating this dilemma is an early detection, diagnosis and eradication of early-stage lesions and their precursors. The use of VELscope^®^ using an autofluorescence as a diagnostic tool might be useful in early detection of oral malignant lesions.

**Materials and methods:**

120 patients with suspicious oral premalignant lesions were examined with two examination methods. They were randomly divided into two groups. Group 1 was examined conventional with white-light and group 2 was examined additionally to the white-light-examination with an autofluorescence visualization device, VELscope^®^. Biopsies were obtained from all suspicious areas identified in both examination groups (n = 52). The diagnostic strategies were compared regarding sensitivity and specificity.

**Results:**

Based upon the result, use of the VELscope^®^ leads to a higher sensitivity (22.0%), but regarding specificity the additional use of the VELscope^®^ is inferior (8.4%).

**Conclusion:**

The VELscope device is a simple, non-invasive test of the oral mucosa, which can help the experienced clinician to find oral precursor malignant lesions.

## Introduction

The increasing prevalence of oral squamous cell carcinomas (OSCC) is a growing problem in many European countries like Germany [1]. OSCC’s may develop from precursor lesions like leuko- or erythroplakias as well as from normal mucosa. The prevalence of leukoplakia differs depending on the country in which it was analyzed. Today, the worldwide prevalence of leukoplakia is approximately 2.6%, while in Germany is 2.3% for men and 0.9% for women. Although it is higher for men in Germany, the potential of malignant transformation is superior in women. The annual rate for a malignant transformation is actually between 1% and 3% [2-4]. It seems therefore reasonable that early evaluation of oral precancerous lesions can have a dramatic impact on oral cancer mortality rates. In this context, the major problem which still remains is the possibility of non invasive chair side, save and reliable differentiation between potentially malignant and non-malignant lesions as well as between “normal” oral mucosa and a micro invasive carcinoma, which can develop out of clinically normal-appearing mucosa [2,5,6]. Current standard diagnostic procedure is the conventional oral examination (COE) under white light conditions with a visual and tactile assessment of the whole oral cavity. This is rather challenging due to lesions, which can occur, as already stated, in clinically normal-appearing mucosa. Hence the surgical biopsy is regarded as the gold standard in this differentiation, even though only 25% of all leukoplakias have been found to be an actual premalignant dysplastic lesions [7]. Epithelial dysplasia is the most important predictor of a malignant transformation and can only be diagnosed in a histological specimen. Beside the histological and clinical side, several chair-side adjunctive aids have been developed to help practitioners with oral cancer screening with the aim of diagnosing high-risk lesions [8]. One alternative to invasive surgical biopsies is the oral brush biopsy that has been intensively assessed in many studies [9]. But by this exfoliative cytology technique it is not possible to analyse deeper cell layers of the oral mucosa in case of lacking minimal invasiveness of the suspected lesion [10]. A completely different potential technique is the use of autofluorescence, which has gained some interest in clinical practice because of the completely non invasive and repeatable character for imaging of the oral mucosa. The use of autofluorescence as a diagnostic tool for cancer detection was first time described as early as in 1924 and was under intensive evaluation for approximately the last 30 years [11]. Autofluorescence uses naturally occurring fluochromes that are located in the epithelium and the submucosa (e.g. collagen, elastin) and which are irradiated with different excitation wavelengths. When irradiated with wavelengths between 375 and 440 nm, the fluochromes show fluorescence in the green spectral range and normal, unaltered mucosa emits a pale green autofluorescence when viewed through a selective, narrow-band filter. A proper filtration is crucial, due to the intense light used for excitation of the fluorochromes. Without a proper filtration, it would be impossible to visualize the pale and narrow autofluorescence signal. However, dysplastic tissues lose fluorescence emission power due to a disruption in the distribution of the fluochromes and appear darker in colour in comparison to the surrounding healthy tissue [12]. The main criticism of autofluorescence in cancer diagnostostics was the lacking ability of discriminating high-risk from low-risk lesions [11]. On the other hand the reliable use of autofluorescence in cancer diagnostics is depending upon a learning curve which means that it requires a lot of experience with the device. Looking at all previous studies there remains uncertainty as to whether the use of adjuncts for identifying and assessing oral mucosal abnormalities results in a meaningful reduction in morbidity and mortality.

The aim of this study was to evaluate the ability of autoflourescence examination to come closer to the answer of this question about its suitability as an adjunct. As an autoflourescence examination handheld device the VELscope (Visually Enhanced Lesion scope; LED Dental Inc., Burnaby, BC, Canada) system was used [13,14] that should prove to delineate between benign, dysplastic and malignant oral mucosa lesions compared to a histological test of the examined tissue.

## Materials and methods

The study was approved by the local ethics committee at the University Medical Centre, Hamburg, Germany (EK37/2011). Study subjects were enrolled in a clinical protocol reviewed and approved by the institutional cancer board. Before the beginning of the study, written informed consent was obtained from each patient.

### Patients

Patients presenting in the department of oral- and maxillofacial surgery in the University Medical Center Hamburg – Eppendorf to rule out invasive squamous cell carcinoma were recruited for this study in a prospective single blinded design. Patients with current advanced squamous cell carcinomas were excluded (Table 1).

**Table 1:** Study inclusion and exclusion criteria

**Inclusion criteria**	**Exclusion criteria**
● Oral premalignant lesion:	● Tumor or tumor recurrences missing operability
● (Leukoplakia, erythroplakia, lichen planus or pemphigus vulgaris)	● Foreseeable missing opportunity of follow-up examination
● Age 18-75	● Pregnancy, heart-, pulmonal-, liver- and kidney disease, chronic pain syndrome nursing, drug addiction, recent operations, and diseases like heart, metabolism, CNS, infectious, circulation, systemic, malignant and immune system affecting diseases as well as blood coagulation disorders and allergic reactions to pharmaceuticals and antibiotics
● Written informed consent	● Dermatological diseases of the face

### Methods

120 patients with suspicious oral premalignant lesions were included for conventional clinical examination and additionally with white-light-examination of oral cancer lesions (COE). They were randomly divided into two groups. Baseline characteristics of the patients are described in Table 2. Group 1 was examined conventional with white-light and group 2 was examined additionally to the white-light-examination with an autofluorescence visualization device, VELscope^®^. In a first step the two groups (only white light vs. white light and VELscope^®^) were compared regarding baseline characteristics to exclude selection bias. Using biopsy as a gold standard, all patients were biopsied. The second step for the group examined with white light and VELscope^®^, the diagnostic strategies were compared regarding sensitivity and specificity.

**Table 2:** Baseline characteristics of group 1 and group 2

	**White light**	**White light plus VELscope**
	**n = 60**	**n = 60**
Age, range	38-82	41-76
Gender, n (%)		
Male	20 (33.3%)	25 (41.7%)
Female	40 (66.7%)	35 (58.3%)
Smoking, n (%)		
Never	10 (16.7%)	7 (11.7%)
Previous	13 (21.7%)	7 (11.7%)
Actual	37 (61.7%)	46 (76.7%)
Alcohol, n (%)		
Never	2 (3.3%)	0 (0%)
≤ 20 g/d	23 (38.3%)	19 (31.7%)
21 – 40 g/d	25 (41.7%)	32 (53.3%)
41 – 60 g/d	6 (10.0%)	4 (6.7%)
61 – 80 g/d	8 (13.3%)	5 (8.3%)
Unknown	0	0
Biopsy taken, n (%)		
Yes	60 (100%)	60 (100%)
No	0	0

The patients had to answer a questionnaire (on a voluntary basis), which included the recent medication and possible cancer risk factors (see Results). In every patient, the lesion was at first clinically examined (COE) by an experienced Oral and Maxillofacial surgeon under “normal” (incandescent) white light and was afterwards examined with the VELscope device. In both examinations, the same areas were observed clinically and with the VELscope device. To exclude the influence of the examiners experience with the device the same examination had been performed by a second surgeon, whereas both did not know about the result of the other. Additionally both examiners were calibrated for the device in advance.

Finally, a surgical biopsy was taken from the respective lesion in local anaesthesia. Written informed consent was obtained from all patients. The participation in this study was voluntarily. A photo documentation was obtained prior to the surgical biopsy with a special objective connecting the camera with the VELscope. All mucosal lesions underwent a histopathological evaluation by an experienced pathologist. The specimens were placed in 4% buffered formalin solution for fixation.

### VELscope device

The machine has been explained in detail in a previous publication [15]. Briefly the VELscope™ (Visually Enhance Lesion scope; MECTRON - European distributor for LED, Vancouver, Canada) device consists of a bench-top casing containing a 120-W metal-halide arc lamp plus a system of filters and reflectors optimized for producing near-UV/blue light between 400 and 460 nm and a coupled handheld unit for direct observation. The device was used under a dimmed room light with protective eye wear worn by the patient throughout the procedure. The autofluorescence excitation device uses visible light in the 430 nm wave length in order to cause fluorescent excitation of certain compounds in the mucosa. According to the existing literature, the complete loss of the normal tissue fluorescence (fluorescence visualization loss) was rated as malignant or dysplastic. A fluorescence in red or orange was not rated as malignant according to the literature [11,14].

### Biopsy preparation

**Figure 1:**
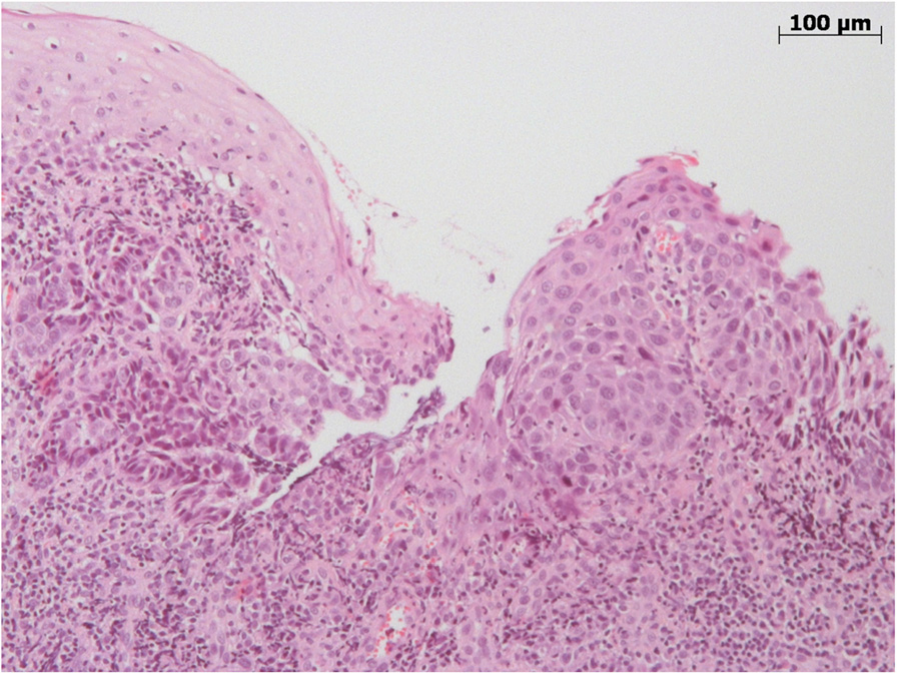
**This representative mucosal biopsy of the oral cavity shows regular squamous epithelium (Figure **1** left side) and a high-grade epithelial dysplasia.** In comparison, the dysplasia on the right hand side of the picture shows a loss of maturation and nuclear atypia, like high nuclear-to-cytoplasmic ratio and nuclear irregularity, involving the whole squamous cell layer of the mucosa. Invasive tumour growth was not found.

All specimens were placed in 4% buffered formalin solution for fixation. Paraffin embedded material was cut into 4 μm thick sections and stained with haematoxlin + eosin (H.E.; MERCK Germany). After color staining all mucosa lesions underwent a histopathological evaluation by two independant experienced pathologist. A representative lesion is shown in Figure 1.

### Statistical analysis

The data collected was analyzed for statistical correlations using IBM SPSS 18 (Statistical Package for the Social Sciences, SPSS Inc., Chicago, IL, USA).

Sensitivity and specificity of the two groups examined with white light and the VELscope device were calculated.

## Results

120 patients with suspicious oral premalignant lesions were randomly divided into two groups. The first group of 60 patients had a conventional examination with white-light. The other 60 patients were examined with an autofluorescence visualization device, VELscope^®^, [AVE] additionally to the white-light-examination. Biopsies were obtained from all patients in both examination procedures (n = 120).

**Figure 2:**
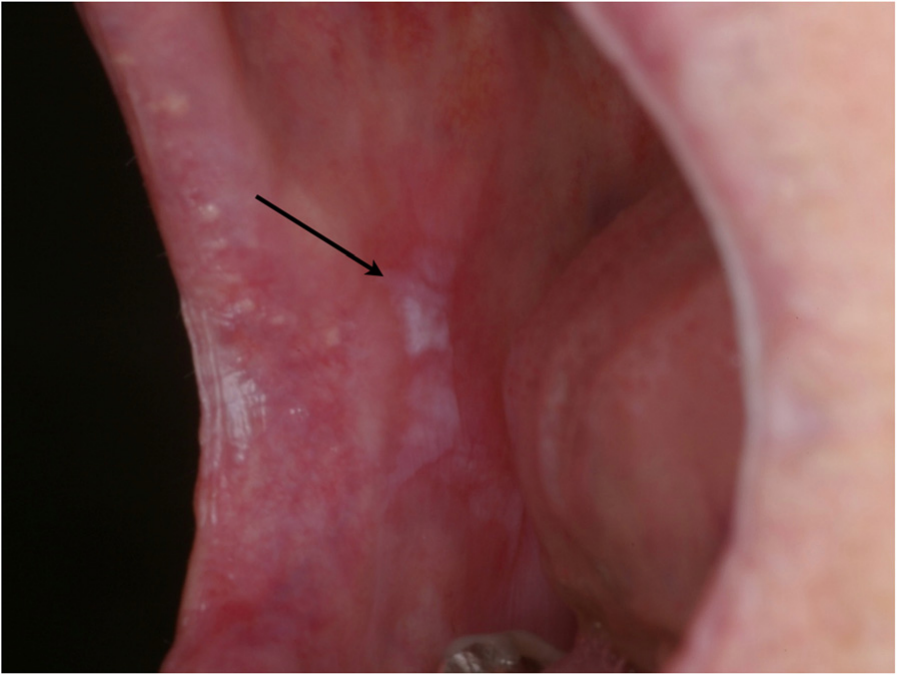
Oral cavity with precancerous lesion of planum buccale.

**Figure 3:**
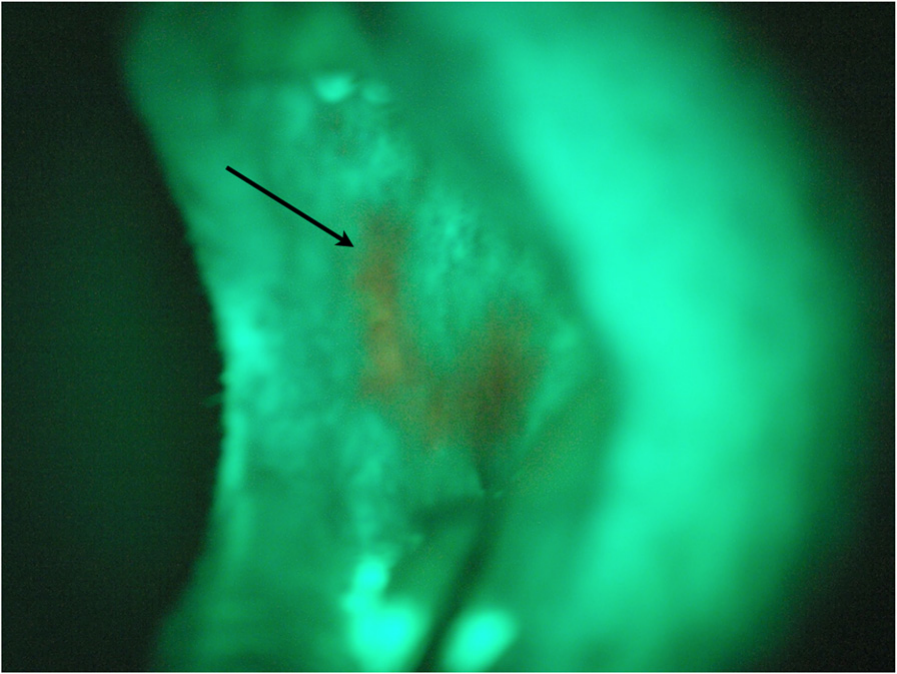
Oral cavity with VELscope examination, the arrow shows the region of loss of fluorescence.

In the first group 45 patients examined with white light were diagnosed positive (Table 3). Out of this 41 biopsies were diagnosed as dysplastic or premalignant lesion (Figures 2 and 3). Table 4 shows the biopsy results of the group examined with the VELscope device. In this group 55 patients were diagnosed positive. Here the biopsies revealed that 47 patients had a premalignant lesion. 5 patients were diagnosed negative, whereas 1 patient had a dysplastic lesion and 4 patients showed no findings.

**Table 3:** Pathohistological findings group 1

	**Pathohistological findings: cancer lesion**	**Pathohistological findings: dysplastic lesion**	**Pathohistological findings: no lesion**
	**n (%)**	**n (%)**	**n (%)**
White light: **positive (n = 45)** n (%)	2 (4.4)	39 (86.7)	4 (8.9)
White light: **negative (n = 15)** n (%)	0 (0)	13 (86.7)	2 (13.3)

**Table 4:** Pathohistological findings group 2

	**Pathohistological findings: cancer lesion**	**Pathohistological findings: dysplastic lesion**	**Pathohistological findings: no lesion**
	**n (%)**	**n (%)**	**n (%)**
VELscope: **positive (n = 55)** n (%)	1 (1.8)	46 (83.6)	8 (14.5)
VELscope: **negative (n = 5)** n (%)	0 (0)	1 (20)	4 (80)

Based on these results the sensitivity, specificity and confidence intervals were calculated. The results are summarized in Table 5. The sensitivity for group 1 is 75.9% and the specificity is 33.3%. The confidence interval for the sensitivity ranges from 65% to 87% and the confidence interval for the specificity ranges from 0% to 71%. The sensitivity for group 2 is 97.9% and the specificity is 41.7%. The confidence interval for the sensitivity ranges from 94% to 100% and the confidence interval for the specificity ranges from 14% to 70%.

**Table 5:** Sensitivity and specificity with according 95% confidence intervals for the two diagnostic procedures

	**Sensitivity**	**95% ci**	**Specificity**	**95% ci**
Difference (white light and Velscope – white light)	22.0%		8.4%	
White light	75.9%	65%–87%	33.3%	0%–71%
White light and VELscope	97.9%	94%–100%	41.7%	14%–70%

## Discussion

The disease specific five-year survival rate especially for patients with advanced oral cancer has not changed over the last decades [16]. It is therefore without question that the most effective way of combating oral cancer is early detection, diagnosis and eradication of early-stage lesions and their precursors [17]. Thus, many oral lesions undergo biopsy only when they display either symptoms or clinical features typical of malignancy, while many innocuous appearing early-stage oral cancerous lesions are merely observed clinically and left undiagnosed. This is especially true for innocuous looking lesions which are subjected to “watchful waiting” and close follow-up despite the fact that some precancerous and cancerous cells within them remain undetected and are allowed to progress to a more advanced stage. It explains the dilemma that more than 50 percent of oral cancers are diagnosed in the advanced stages. Early detection of oral premalignant or cancerous lesions is still a problem. Visual inspection under white light often cannot differentiate between lesions harboring dysplasia and/or early cancer from those that do not. The practice of not properly evaluating all suspicious lesions, that is, lesions without a specific etiology such as trauma or infection, invariably results in delay of the correct diagnosis, limiting treatment options. In contrast to tissue alterations in other sites of the body subtle changes of cytologic abnormalities in oral mucosa are more difficult to obtain [18]. Methods and tools which improve the diagnostic abilities of primary care dentists/physicians that are also less invasive than biopsies and better than interventional or imaging investigations in secondary care still remain important points for research in this field [11]. In this study, we presented the use of autofluorescence as a diagnostic tool and evaluated its accuracy in the detection of oral malignant lesions. The autofluorescence findings with the VELscope device were compared to the clinical findings and the histological evidence of biopsies taken from the region of interest. In contrast to other studies our collective comprised also patients having had oral cancer treatment including radiation therapy in history. On the one hand this makes the collective more inconsistent, on the other hand it demonstrates the properties of the VELscope device in alterated mucosa which makes clinical examination even more difficult. On the other hand taking a biopsy is more invasive in a preradiated patient than in normal healthy tissue.

The statistical analysis showed, due to the relative small number of patients with SIN or invasive carcinomas a therefore limited specificity (80,8%) and high sensitivity (100%) within this trial. These findings are comparable to the data range in the current literature [2,11,12,15,16]. One study was also performed in a single blinded fashion like ours showing 77%/95% specificity/sensitivity. However, it should be kept in mind, that these results were obtained from a high-risk patients group with some having had a previous oral malignant neoplasm in history. All patients were referred to our department to rule out a malignant disease because a primary care physician evaluated the lesions to be suspicious. Consequently, the results cannot be transferred to the chair side screening situation in a general population. This circumstance already “occurred” in many other studies in the literature [2,11,12,15,16] and have to be considered as a drawback of this and any other study of the main part of the existing literature covering this topic. Some previous studies were conducted in patients with known oral dysplasia or SCC confirmed by biopsy and did not involve use of the technology as an adjunct for detection or diagnosis of new lesions [19-21]. To our knowledge, only few trials observed the ability of autofluorescence as a diagnostic tool in clinically unaltered mucosa [8,17,22,23], but the results represent anecdotal observations, due to very low case numbers. In these studies, VELscope was unable to discriminate between dysplasia and non-dysplasia cases in 13 cases. Therefore these preliminary findings have to be further investigated in the form of controlled prospective studies. But any further study evaluating the latter field has to answer the question whether the use of adjuncts for identifying and assessing oral mucosal alterations results in a meaningful reduction in morbidity and mortality.

The unnecessary taken biopsies would also increase the morbidity risks in the context of these procedures. In contrast, there are several studies, which underline the ability of the device to identify areas of dysplasia [9,19]. All in all, recent studies from Awan *et al.* and Rana *et al.*[11,15] showed comparable data within high-risk groups with high sensitivity and relatively low specificity. This lack of specificity remains a constant problem in clinical routine use and was also one of the main drawbacks of all studies [11,12,24,25]. Because of this low specificity the autofluorescence examination could lead to overdiagnosis in the hands of general practitioners. From our point of view, the use of the VELscope device is a highly subjective examination and strongly depends on the experience of the individual examiner with the device and the clinical estimation of any oral cancerous lesions in general. Therefore all oral maxillofacial surgeons having participated this study underwent a training on minimum ten oral lesions with three repetitions on different time points training the VELscope machine prior to this clinical trial. Consequently, in clinical practice the low specificity lead to a high amount of unnecessarily taken biopsies. This was also seen in recent publications covering this topic [11,12,26]. Any permanent mucosal lesion that does not have an obvious etiology such as trauma or infection needs further diagnostic. Failure to conform to the standard of care, which requires all unexplained lesions to be evaluated, can have dire consequences for both the patient and the oral care provider [27,28]. The results of this longitudinal study, matching fluorescent light diagnostic and scalpel biopsies were performed simultaneously on patients with minimally suspicious oral lesions demonstrate that the VELscope device is a highly sensitive and specific, noninvasive test in the chair side evaluation of all oral lesions without an etiology. The device is even more beneficial when used on lesions that appear clinically benign for identifying early stage cancers and dysplasias - the lesions for which therapy is most effective. As a non invasive adjunct to routine oral cancer examination, its use has the potential to reduce the poor mortality rate associated with oral malignancies.

## Conclusion

The VELscope device is a simple, non-invasive test of the oral mucosa, which can help the experienced clinician to find oral precursor malignant lesions and the correct location for taking biopsies within the altered mucosa. Nevertheless, the results should be interpreted with caution due to the issue of frequently occurring false positive results. The device should not be used in the hands of unexperienced clinicians and cannot be a replacement for the gold standard of any histological evaluation. At the moment the device can only be recommended to exclude any suspicious lesion.

### Consent statement

Written informed consent was obtained from the patient for publication of this case report and accompanying images. A copy of the written consent is available for review by the Editor-in-Chief of this journal.

## Competing interests

The authors declare that they have no competing interest.

## Authors’ contribution

HH, JK, RS, MH, MB, WE, TC, AG, AK and MR conceived of the study and participated in its design and coordination. JK and HH made substantial contributions to conception and design of the manuscript as well as data acquisition. MH, MB, TC, AG have been involved in drafting the manuscript. RS and MR were involved in revising the manuscript. All authors read and approved the final manuscript.
